# Observations on the Urine

**Published:** 1803-04-01

**Authors:** 

**Affiliations:** Liverpool


### Observations *on the* Urine ; *communicated by* Mr. Bostock, *of Liverpool.*

It has been remarked by one of the French chemists, that no substance has given rise to a greater variety of experiments, or been productive of more important discoveries, than the urine. The number of the ingredients which enter into its composition, the singularity of its products, the complicated nature of the spontaneous changes which it undergoes, and the varieties to which it is subject, as connected with the state of the living system, have long caused this fluid to be an object of great attention both to the chemist and the physician. But to the same circumstances which have rendered it the object of so much attention, must in part be ascribed the imperfect and confused ideas which were so long entertained respecting its nature and composition ; even in the present day, the most accurate chemists are not entirely agreed in their accounts of its constituents, or of the changes which are effected by their spontaneous action upon each other. Several very interesting particulars respecting the nature and composition of the urine were discovered by the celebrated Scheele ; and since his time, Fourcroy has applied himself with extraordinary assiduity to the examination of this fluid. See Ann. de Chimie, t. 31. An elaborate account of it has been published by this author in his recent work, entitled Systeme des Connoissances Chimiques. A valuable Treatise on this subject has also appeared from the pen of Mr. Cruickshanks ;* it is rendered peculiarly useful to the experimenter, from a correct account of the different re-agents, by means of which the varieties that take place in the nature and quantity of its impregnations may be easily detected. It will however be found, upon comparison, that the analyses of Fourcroy and Cruickshanks do not in every particular coincide ; and it seemed of some importance that these differences should be accurately detailed, in order that they may be decided by future experiments. The well established facts are arranged in a method, which appears clear and perspicuous ; and those parts of the subject are pointed out, which seem to require the aid of further experiment, in order to their complete investigation.

The

* Rollo on Diabetes, and Thomson's Fourcroy, vol. iii. p. 315.

The urine may be considered under the five following heads. 1st. Its state immediately after being voided by the bladder, when it proceeds from a healthy subject, and before it has undergone any alteration. 2d. Its state after its component parts have undergone a change, but before the putrefactive fermentation has taken place. 3d. Its state after it has become putrid. 4th. The substances which are occasionally found in it, when there is no disease in the system, but which are not essential to its existence as urine. 5th. Its state when the system is affected by disease.

Sect. 1. The state of the urine immediately after being voided from the bladder.

According to Fourcroy, recent, healthy urine contains the following substances held in solution. 1st. A peculiar matter, upon which most of the distinguishing properties of the urine depend, called by Fourcroy and Vauquelin, who first distinctly ascertained its nature, Urée. 2d. Gelatine; the existence of the gelatine in urine was first discovered by Seguin, in consequence of the addition of the tanning principle. 3d. Muriat of soda. 4th. Muriat of ammoniac; this exists in but small quantity, and is so intimately united to the urée, that it is almost impossible entirely to separate them. 5th. Phosphat of soda; this was for a long time confounded with the phosphat of ammoniac. 6th. Phosphat of ammoniac; this is the salt to which the greatest attention has always been paid by chemists, as it is that from which phosphorus is obtained in the usual processes; it is always joined to the phosphat of soda, and there appears to be a ternary compound formed by their union; this compound has obtained the name of microcosmic salt. 7th. An acidulous phosphat of lime; phosphat of lime is insoluble in water, but when added to an excess of acid, as it exists in the urine, it becomes soluble. 8th. Phosphat of magnesia; this salt is only found pure in urine which is perfectly recent; it afterwards becomes united with ammoniac, and forms with it a triple salt. 9th. The phosphoric acid in excess, which gives solubility to the phosphat of lime, and causes recent urine to possess acid qualities. 10th and 11th. Small quantities of uncombined lithic, or, as it is now called, uric acid, and also of the benzoic acid; Scheele first detected the presence of these two substances in the urine.

Fourcroy describes at considerable length the nature and properties of the urée. If the urine be gently evaporated to the consistence of a thick syrup and then cooled, cooled, a mass will be formed which consists of all the solid contents of the urine. The urée is found to be by far the most considerable of these, composing in some instances nineteen parts out of twenty of the weight of the mass. It is soluble in about four times its weight of alcohol; and if the alcohol be driven off by distillation, the urée will be procured nearly pure; a small quantity of muriat of ammoniac and of Benzoic acid alone will be left adhering to it: these are the only salts of the urine upon which alcohol has any action. If the urée be distilled, and the products be received in the pneumatic apparatus, we shall find it to consist of azote, hydrogen, carbon, and oxygen; of these, the azote is in the largest proportion. A watery solution of pure urée may be kept for some time without any change taking place in it; but if an animal substance be added, decomposition commences in a few days, and there is a formation of the carbonic and acetous acids and of ammoniac: this decomposition in the urine is effected by the presence of the gelatine. If a solution of the urée in water be distilled, and more water be continually added to the residue, it is converted almost entirely into the carbonat of ammoniac: it is the only animal substance which is decomposed at the temperature of boiling water. If diluted nitric acid be digested upon a thick watery solution of urée, a large quantity of lamellated crystalline matter will be produced, which Fourcroy supposes to consist of urée united to the nitric acid. Ammoniac has no action upon this substance; soda, pot-ash, barytes, and lime, all decompose it: a minute account of the products may be found in Fourcroy. Urée possesses the singular property of entirely changing the form of the crystals of muriat of soda and of ammoniac; it is yet uncertain whether it extends this effect to any other of the neutral salts: this substance is more readily dissolved in hot than in cold alcohol; it is precipitated in a crystalline form, by cooling a saturated solution of it in hot alcohol.

Mr. Cruickshanks's analysis of recent urine differs in some respects from Fourcroy's. The former chemist enumerates the following substances as existing in the residuum produced by evaporation: "The muriats of pot-ash and soda, the phosphats of lime, soda, and ammoniac, the phosphoric and lithic acids, with animal, extractive matter." Mr. Cruickshanks seems not to have been fully aware of the peculiar nature of the animal extractive matter, as he says, that it yields by distillation "nothing more than the usual products from animal substances." He details tails at length the action of the diluted nitric acid upon it, and describes the lamellated crystalline matter which is thus produced; but he differs from Fourcroy respecting its nature, as he conceives it to be "an animal acid hitherto unknown, whose base exists in this extractive matter."—Cruickshanks estimates the quantity of gelatine discoverable by the action of tannin, to be about 1 grain in 1 oz. of healthy urine. Fourcroy considers the muriat of potash as a substance which is only occasionally detected in the urine; but Cruickshanks, on the contrary, conceives that this salt is always present, and generally in greater plenty than the muriat of soda. In the analysis of Cruickshank, we find no mention of the phosphat of magnesia, nor of the Benzoic acid, the former of which substances appears to have been first detected in the urine by Fourcroy and Vauquelin, the latter by Scheele.

Sect. 2. The state of the urine after its component parts have undergone a change, but before the putrid fermentation has commenced.

One of the first changes which takes place in urine is the deposition of a red powder, which is found to be the uric acid; it is separated, in part at least, during the cooling of the urine, as it is much more soluble in hot than in cold water. Another change which soon begins to be produced, is the loss of its acid properties; this depends upon the generation of ammoniac, which saturates the excess of phosphoric acid; the phosphat of lime is therefore no longer soluble, but is precipitated in the form of a whitish powder. The production of ammoniac continues to increase, and besides saturating the phosphoric, uric, and benzoic acids, it is in part united to the phosphat of magnesia and produces with it a triple salt. This magnesian phosphat of ammoniac, not being soluble in water, is also deposited. When the urine has advanced to this state, its sensible properties and general appearance become much altered, and the production of carbonic acid announces that the complete putrefactive fermentation has taken place.

Sect. 3. The state of the urine after it has become putrid.

The peculiar animal matter to which Fourcroy and Vauquelin have given the name of Urée, is the substance upon which most of the changes depend, both in urine when recently voided, and in this fluid when it has acquired the putrid state. The facility with which it is decomposed, forms one of its distinguishing characters; and Fourcroy has discovered, that the heat of boiling water is sufficient to to convert it almost entirely into the carbonat of ammoniac. Animal bodies, in general, show a greater readiness to decomposition than vegetable substances, and this property appears to be in proportion to the quantity of azote which they contain; the urée, as was remarked above, contains an unusually large proportion of this element. The first operation of the urée is the production of ammoniac, by which the first sets of changes are produced. In addition to the ammoniac, the urée now begins to generate carbonic acid; and this coming into contact with the alkali immediately upon being produced, is neutralized, and a carbonat of ammoniac is formed. As the decomposition of the urée becomes more complete, acetous acid is also evolved; and this, as in the former case, is converted into the acetit of ammoniac. While these several changes are going forwards, the fluid becomes entirely altered in its colour, smell, and other sensible properties, and will also be found to differ very materially from recent urine in its chemical constituents. The following are the ingredients of putrid urine: 1st. A portion of urée, still undecomposed; 2d. Ammoniac in excess; 3d. Phosphat of ammoniac; 4th. Magnesian-phosphat of ammoniac; 5th. Urat of ammoniac; 6th. Acetit of ammoniac; 7th. Benzoat of ammoniac; 8th. Muriat of ammoniac; 9th. Carbonat of ammoniac; 10th. Muriat of soda; 11th. Ternary, composed of phosphat of soda and ammoniac. The gelatine is at first precipitated, and afterwards probably decomposed; the phosphat of lime is also precipitated.

Sect. 4. Substances which are occasionally found in urine.

In this class Fourcroy ranks the following bodies: Muriat of pot-ash, sulphat of soda, oxalat of lime, albumen, and silex. To these may be added a variety of substances which are immediately derived from the food; the specific odour of many vegetables is perceived in the urine soon after they have been employed as articles of diet or medicine; and after the use of iron and nitre, they have been detected in the urine by their appropriate tests. The colouring matter of rhubarb and madder are also carried out of the body by the urinary organs; and from the effects produced, we may conclude that the irritating part of cantharides is conveyed to the bladder unchanged through the course of the circulation. It is probable, that as experiments are multiplied upon the urine, still more extraneous substances will be discovered there; its visible appearance and smell are known to be affected in some measure sure by almost all the substances that are received into the stomach.

Sect. 5. The state of the urine when the system is affected by disease.

The varieties which are produced in the urine by different diseases have attracted the attention of mankind from the earliest ages; but, owing to the imperfect state of chemical knowledge, little accurate information has been obtained respecting the nature or causes of these changes. Fourcroy enumerates the following different species of urine, each of which is produced by some specific morbid state of the body; the inflammatory, the bilious, the critical, the nervous, the gouty, the calculous, the rickety, and the diabetic; to which, according to the observations of Mr. Cruickshanks, we may add the urine of dropsical patients; future observations will probably enlarge the list. It must indeed be acknowledged, that little improvement either in the theory or practice of medicine has hitherto been derived from the examination of the urine; but this may be ascribed to the imperfect and even erroneous ideas that have been entertained respecting this fluid. The urine of diabetes is that species on which the most numerous and accurate experiments have been made, and the singular fact of the production of saccharine matter, in every respect similar to the sugar of vegetables, is established beyond a possibility of doubt. Yet the analysis of diabetic urine is still very imperfect; we are ignorant what quantity of ureé it contains, or whether it is similar to this substance as it exists in healthy urine; and the nature and properties of its saline impregnations are still undetermined. The spontaneous changes which it undergoes, have hitherto been examined only in a vague and cursory manner, and little is yet known respecting the operation of the different chemical re-agents upon it. We are little acquainted with the chemical nature of either *inflammatory* or *critical* urine. They are known to deposit a large quantity of a reddish sediment, which has been supposed to consist of phosphat of lime mixed with the uric acid. Mr. Cruickshanks informs us, that oxygenated muriat of mercury, when thrown into the urine of patients, with an inflammatory action of the vessels, causes a milkiness, and throws down a white precipitate. It has been generally imagined that *bilious* urine differs from healthy urine solely by the addition of a quantity of bile which is dissolved in it; Fourcroy however is disposed to doubt whether this is actually the case. *Nervous* urine consists consists probably of the usual constituents, only diluted with a much larger proportion of water. A copious sediment of the phosphat of lime is generally observed in the urine of *gouty* persons; Berthollet has discovered that their urine contains less than the usual proportion of uncombined phosphoric acid, which in healthy urine keeps the phosphat of lime suspended; before the accession of a paroxysm he has observed the acid quality of the urine to disappear altogether. Notwithstanding the numerous experiments which have been made upon the subject, we are still unable to ascertain the precise nature of *calculous* urine. Urinary calculi certainly differ very materially from each other in their chemical nature; and, probably, the urine from which they are formed would have been found equally various in its composition. It is probable that in these cases there is an excess of animal matter, but it still remains to be determined whether this is albumen, gelatine, or urée. The uric acid constitutes a considerable proportion of at least the greatest number of calculi, and in these instances we may conjecture that this acid exists in unusual quantity in the urine. Oxalat of lime, silex, and other substances, have occasionally been found in calculi; but these are to be regarded as accidental, and are not to be considered as essential to calculous urine. In some cases they appear to act merely mechanically, by affording a solid centre about which the other substances can more conveniently concrete. The large quantity of sediment which is deposited from the urine of *rickety* children, is generally considered to consist of the phosphat of lime proceeding from the decomposition of their bones, an effect which has been ascribed to the generation of oxalic acid in the system. Respecting *dropsical* urine, we are informed by Cruickshanks that nitric acid, the oxygenated muriat of mercury, and alum, produce a precipitation which appears to be of a serous nature.

This general view of the different kinds of morbid urine will probably be found to contain all the well ascertained facts upon the subject; and while it shows the very imperfect state of our knowledge respecting the diseases of this fluid, it strongly points out the necessity of a more accurate investigation of this important part of physiology.

*March* 10, 1803.

